# Elucidating Hedgehog pathway's role in HNSCC progression: insights from a 6-gene signature

**DOI:** 10.1038/s41598-024-54937-6

**Published:** 2024-02-26

**Authors:** Yang Yang, Chenxi Yang, Qiying Yang, Shun Lu, Bisheng Liu, Dongyun Li, Dongliang Li, Peng Zhang, Peng Xu, Jinyi Lang, Jie Zhou

**Affiliations:** 1 Department of Oncology, The Third People’s Hospital of Chengdu, Chengdu, 610014 China; 2 Department of Radiation Oncology, Radiation Oncology Key Laboratory of Sichuan Province, Sichuan Clinical Research Center for Cancer, Sichuan Cancer Hospital & Institute, Sichuan Cancer Center, Affiliated Cancer Hospital of University of Electronic Science and Technology of China, Chengdu, 610042 China; 3 Military Casualty Management Department, General Hospital of the Western War Zone of the Chinese People’s Liberation Army, Chengdu, 610036 China

**Keywords:** Hedgehog pathway, Prognosis, Immunotherapy, Tumor microenvironment, Head and neck squamous cell carcinoma, Cancer, Head and neck cancer

## Abstract

With the emergence of targeted inhibition strategies for Hedgehog signaling in cancer, multiple Hedgehog signaling pathway-related biomarkers have become the focus of research. SsGSEA algorithm was employed to analyze the Hedgehog pathway scores of samples in TCGA-HNSC dataset and divide them into two groups. Weighted co-expression network analysis was performed to identify modules strongly associated with the Hedgehog pathway. Differentially up-regulated genes in tumor samples in comparison to the normal ones were screened by Limma, in which genes belonging to modules strongly related to Hedgehog pathway were further filtered by LASSO reduction and multivariate Cox regression analysis to develop a model. ESTIMATE and CIBERSORT were served to characterize the tumor microenvironment (TME). TIDE assessed immunotherapy response. Hedgehog pathway activity was significantly higher in head and neck squamous cell carcinoma (HNSCC) tissues than in normal tissues and was correlated with HNSCC survival, glycan, cofactors and vitamins, drug metabolism, and matrix scores. Six genes (SLC2A3, EFNB2, OAF, COX4I2, MT2A and TXNRD1) were captured to form a Hedgehog associated 6-gene signature, and the resulting risk score was an independent indicator of HNSCC prognosis. It was significantly positively correlated with stromal score, metabolism, angiogenesis and inflammatory response. Patients in low-risk group with a low TIDE score had higher immunotherapy sensitivity relative to those in high-risk group. This study revealed novel findings of the Hedgehog pathway in HNSCC progression and opened up a Hedgehog pathology-related signature to help identify risk factors contributing to HNSCC progression and help predict immunotherapy outcomes.

## Introduction

Head and neck cancer as one of the common cancers with a high incidence worldwide consists of a variety of tumors affecting the upper respiratory gastrointestinal tract, including the tongue, mouth, lips, nasal cavity, nasopharynx, oropharynx, paranasal sinus, larynx, and salivary glands^1–3^. HNSCC is the most common type of head and neck cancer, roughly 60% of HNSCC cases could be classified as locally advanced at time of diagnosis^4,5^. Open and minimally invasive surgery is standardized treatment for most patients with oral and early laryngeal cancers, whereas the rest HNSCC cases are treated by radiotherapy or concurrent chemoradiotherapy^6,7^. The recurrence rate of HNSCC after initial treatment can reach 70%, and the majority of cases are reported to have a poor prognosis^8^. Challenges remain in all aspects of HNSCC management. Molecular genetic landscape of HNSCC has revealed new possibilities for clinical therapeutic intervention, leading to an increasing awareness of the significance of modifiable risk factors.

Hedgehog signaling is switched off to remain quiescent in adults and remains active in stem cells in the skin, central nervous system, and gut to maintain tissue homeostasis and regeneration^9^. During the initiation and development of cancer, hedgehog pathway is abnormally activated and participates in the induction of malignant phenotype, including the promotion of proliferation, the promotion of invasion, metastasis, angiogenesis and tumor inflammation and the inhibition of cell death signals, as well as the dysregulation of cell metabolism^9,10^. Targeted inhibition of Hedgehog signaling has been exploited as a novel strategy for treating various cancers that block hedgehog signaling at different steps, including Hgengehog acetyltransferase inhibitors, small-molecule Hedgehog inhibitors, Smo inhibitors, and Gli inhibitors^11^. Current FDA-approved Hedgehog signaling inhibitors include vismodegib^12^, sonidegib^13^ and glasdegib^14^. All three compounds inhibit hedgehog signaling by targeting SMO^15,16^. Drug-resistant mutations and SMO-independent hedgehog activation limit the clinical applicability of SMO antagonists. The development of multi-targeted Hedgehog inhibitors becomes a promising approach in the future^17^.

A basic understanding of the mechanisms involved in hedgehog signaling is necessary to improve tumor suppression^18^. The current study focused on exploring the impact of Hedgehog pathway on the pathological process of HNSCC. We created a multi-target model associated with Hedgehog pathway and compared it with clinical features to identify a more scientific hierarchical management strategy for understanding the metabolism, tumor microenvironment (TME), immunotherapy response, and prognosis of HNSCC.

## Materials and methods

Transcription profiles and clinicopathological data were collected from the TCGA-HNSC cohort from the Cancer Genome Atlas (TCGA; https://portal.gdc.cancer.gov) and the GSE65858 and GSE41613 cohorts from Gene Expression Omnibus (GEO; http://www.ncbi.nlm.nih.gov/geo). The data of individuals with clinical information such as age and gender, as well as overall survival (OS) and survival status were extracted, including 491 individuals in the TCGA-HNSC cohort, 251 individuals in the GSE65858 cohort, and 96 individuals in the GSE41613 cohort. FPKM provided by TCGA-HNSC was converted to TPM, then log2(TPM + 1) was calculated and Ensembl was converted to Gene symbol.

### Download of metabolic pathways and calculation of enrichment scores in samples

The HALLMARK pathways were downloaded from MsigDB database^19^, and the keggGet function of KEGGREST package was served to download the list of genes contained in these pathways according to the pathway number provided by Nucleotide metabolism, Energy metabolism, Carbohydrate metabolism, Metabolism of other amino acids, Xenobiotics biodegradation, Amino acid metabolism, Lipid metabolism, Metabolism of terpenoids and polyketides, Metabolism of cofactors and vitamins, Biosynthesis of other secondary metabolites, metabolism, and Glycan biosynthesis and metabolism, Chemical structure transformation maps in KEGG website (https://www.kegg.jp/). Based on the above gene sets, we performed a single-sample gene set enrichment analysis (ssGSEA) using the R package GSVA. Normalization was first performed, with standard quantile normalization methods used. The gene set enrichment scores were then calculated for each sample.

### Weighted co-expression network analysis (WGCNA)

WGCNA package^20^ of R software was used to develop a co-expression network, and two files were used, one was gene expression file and the other was phenotype file. The Median Absolute Deviation (MAD) of all protein-coding genes in the TCGA-HNSC cohort was calculated across samples. The top 5000 genes with the largest MAD were considered as potential dysregulated genes and were used for WGCNA analysis. The phenotype file prepared was the Hedgehogs pathway. In the analysis process, the pickSoftThreshold function calculated the soft threshold conforming to the standard of scale-free network, and converted the similarity matrix to the adjacency matrix and then to the topological overlap matrix (TOM). Dynamic tree cutting method was used for module identification, and the relationship between each module and Hedgehogs pathway was calculated and visualized.

### Establishing of Hedgehog-associated signature

Limma^21^ analysis in R was served to screen differentially up-regulated genes in tumor samples compared with normal samples in the TCGA-HNSC cohort, with a threshold of (log2(FC)) > log2(1.5) and FDR < 0.05. Lasso regression is a machine learning algorithm that adds regular term on the basis of general linear regression to ensure the best fitting error, and ensure that the parameters are as simple as possible, so that the model has strong generalization ability. For the LASSO COX analysis, the penalty parameter λ was selected by fivefold cross-validation and candidate genes with non-zero coefficients were obtained. The intersection genes between the target differentially up-regulated genes and the genes in the Hedgehogs pathway-related modules were selected for LASSO reduction and multivariate Cox regression analysis to construct a Hedgehog associated signature. Z-score based on risk score predicted prognostic status and was observed by plotting Kaplan–Meier curves. The prognostic classification efficiency of Hedgehog associated signature was evaluated by ROC analysis performed with the R package “timeROC”^22^.

### Construction and evaluation of the nomogram

The independence of clinicopathological parameters and risk score in predicting HNSCC prognosis was assessed by performing univariate and multivariate Cox regression analyses were performed to determine. Indicators that were confirmed as independent prognostic factors were imported into “Rms” to generate a nomogram. The accuracy and validity of the Nomogram were evaluated by drawing the calibration Curve and Decision Curve Analysis (DCA) curve.

### Functional enrichment analysis

Functional enrichment analysis was performed using two packages, ssGSEA of the GSVA package and clusterProfiler^23^. The pathways used for ssGSEA were downloaded from KEGG and included TGF-beta signaling pathway, VEGF signaling pathway, Wnt signaling pathway, Hedgehog signaling pathway, VEGF signaling pathway, and Notch signaling pathway. The gene list file and reference gene set were read, and the enrichment analysis and visualization were performed by running ClusterProfiler.

### Immunologic analysis

Different metrics of tumor immunology, including immune cell infiltration, tumor immune circulation, immunophenotypic scores, and immunotherapy response, were analyzed using different algorithms. CIBERSORT applies LM22 as the reference data set and outputs the proportion of immune cell infiltration based on the reference data set according to the sequencing expression matrix provided^24^. Immune cell infiltration of samples in the TCGA-HNSC cohort was also calculated using 29 functional gene expression signatures (Fges) representing the major provided in published literature functional components and TME provided in published literature^25^. A one-stop web-based tool, TIP, was used to analyze and visualize the activities of seven-step anti-cancer immunity steps^26^. The immunophenoscore (IPS) quantitative scoring scheme developed by Charoentong et al. was used to determine the impact on tumor immunogenicity. IPS were calculated on an arbitrary scale of 0–10 according to the sum of the weighted average z-values of the four categories^27^. TIDE scores were calculated for each tumor sample with the use of “Predict Response” function of TIDE (http://tide.dfci.harvard.edu/) as a predictor of immune checkpoint blockade (ICB) response.

### Statistical analysis

Data processing, statistical analysis and visual analysis of the results were done in R software. Student’s *t* test was served to compare the differences between the two groups of variables conforming to normal distribution. Wilcoxon rank sum test was utilized to analyze statistical significance between the two groups of non-normally distributed variables. P values marked directly or expressed by */ns, *P < 0.05, P < 0.01, ***P < 0.001, ****P < 0.0001.

## Results

### Hedgehog pathway was overactive in HNSCC and associated with prognosis and metabolism

**Figure 1:**
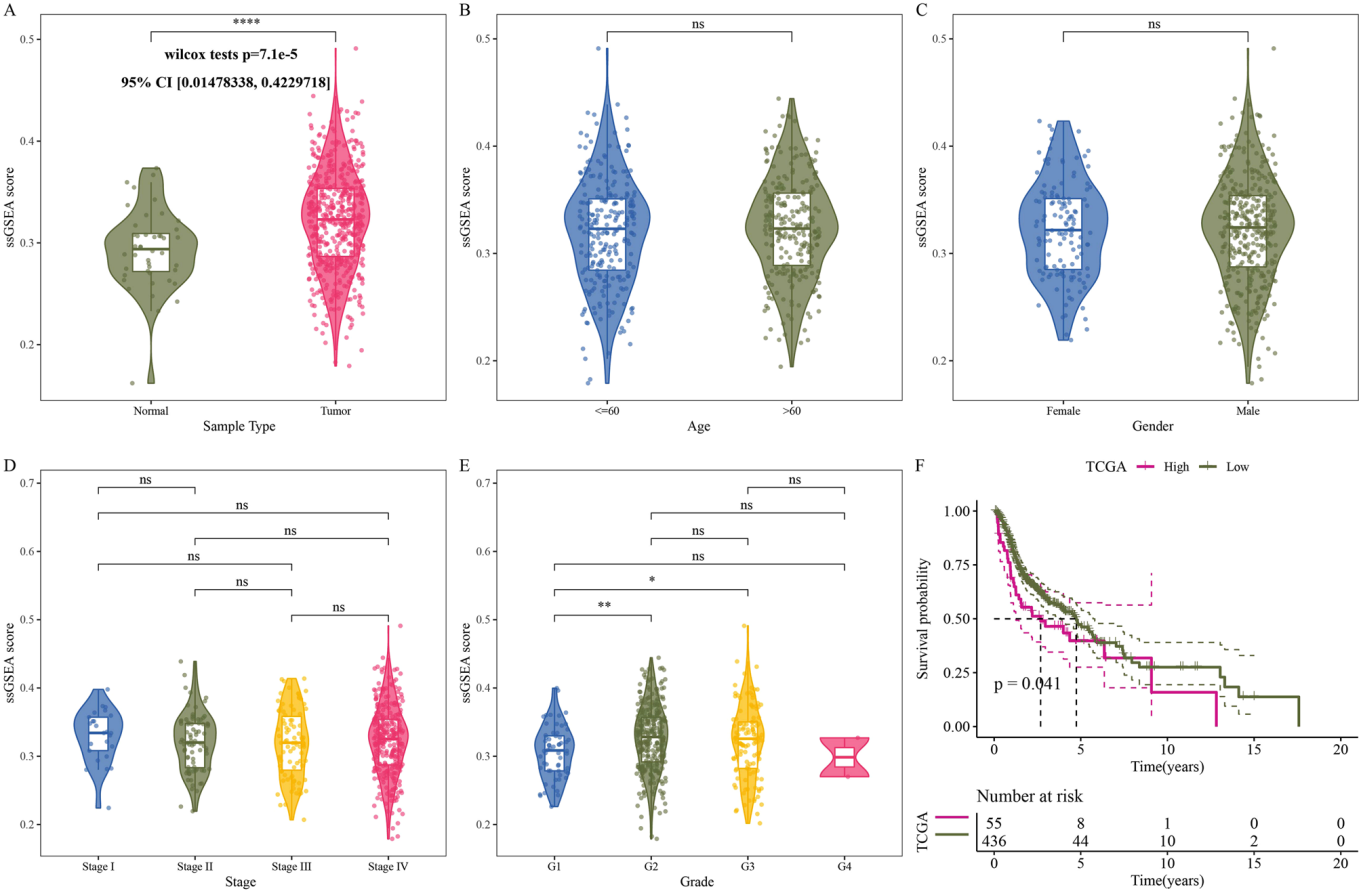
Hedgehog pathway was overactive in HNSCC and associated with prognosis and metabolism. (**A**) Hedgehog pathway enrichment scores for normal and tumor samples in the TCGA-HNSC cohort. (**B**) Hedgehog pathway enrichment scores for the two age groups grouped with a cutoff of 60 years. (**C**) Differences in Hedgehog pathway enrichment scores between male and female samples. (**D**) Hedgehog pathway activity between the four stages. (**E**) Hedgehog pathway activity of different grade. (**F**) Kaplan–Meier curves for samples plotted based on Hedgehog pathway scores.

**Figure 2:**
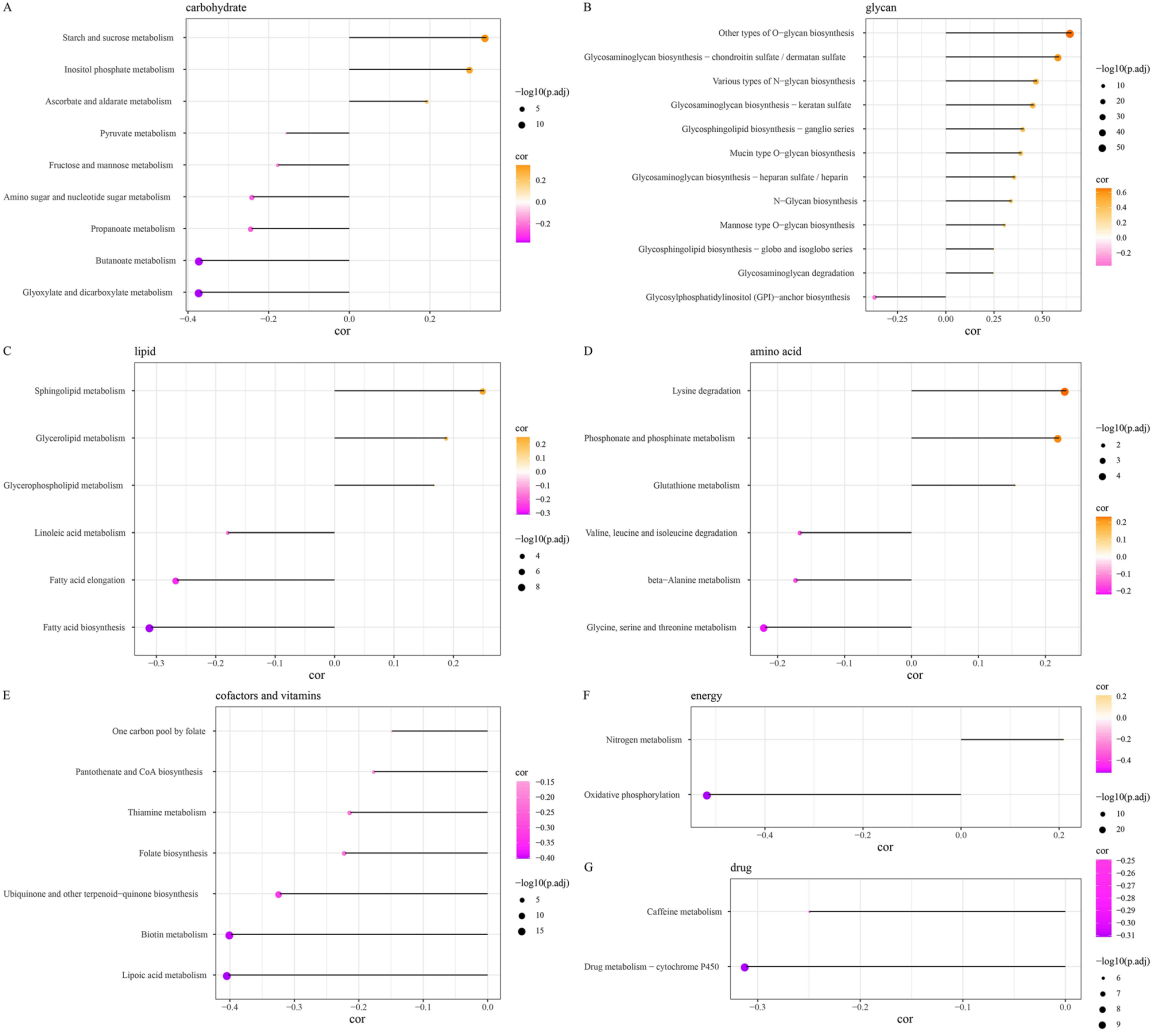
Correlation between Hedgehog pathway scores and different metabolism in HNSCC.

We analyzed Hedgehog pathway activity between tumor and normal samples in TCGA-HNSC cohort and found that tumor samples showed significantly enhanced Hedgehog pathway activity compared with normal samples (Wilcox tests p = 7.1e−05, 95% CI: [0.01478338, 0.04229718]) (Fig. 1A). The activity of Hedgehog pathway did not change significantly with the passage of age and stage, and had no significant correlation with gender (Fig. 1B–D). Hedgehog pathway activity in G2 and G3 was significantly higher than that in G1 (Fig. 1E). Hedgehog pathway scores showed a significant correlation with 5-year survival, with samples with low Hedgehog pathway scores showing preferential survival over samples with high Hedgehog pathway scores (Fig. 1F). Therefore, the Hedgehog pathway may be a potential factor for poor prognosis in HNSCC patients. The correlation between Hedgehog pathway activity and metabolic pathways, including glycan metabolism, carbohydrate metabolism, lipid metabolism, amino acid metabolism, cofactors and vitamins metabolism, energy metabolism and drug metabolism, was analyzed. Hedgehog pathway activity was significantly positively related to most glycan metabolism, cofactors and vitamins, and drug metabolism (Fig. 2).

### The Hedgehog pathway showed a potential impact on the TME of samples in HNSCC

**Figure 3:**
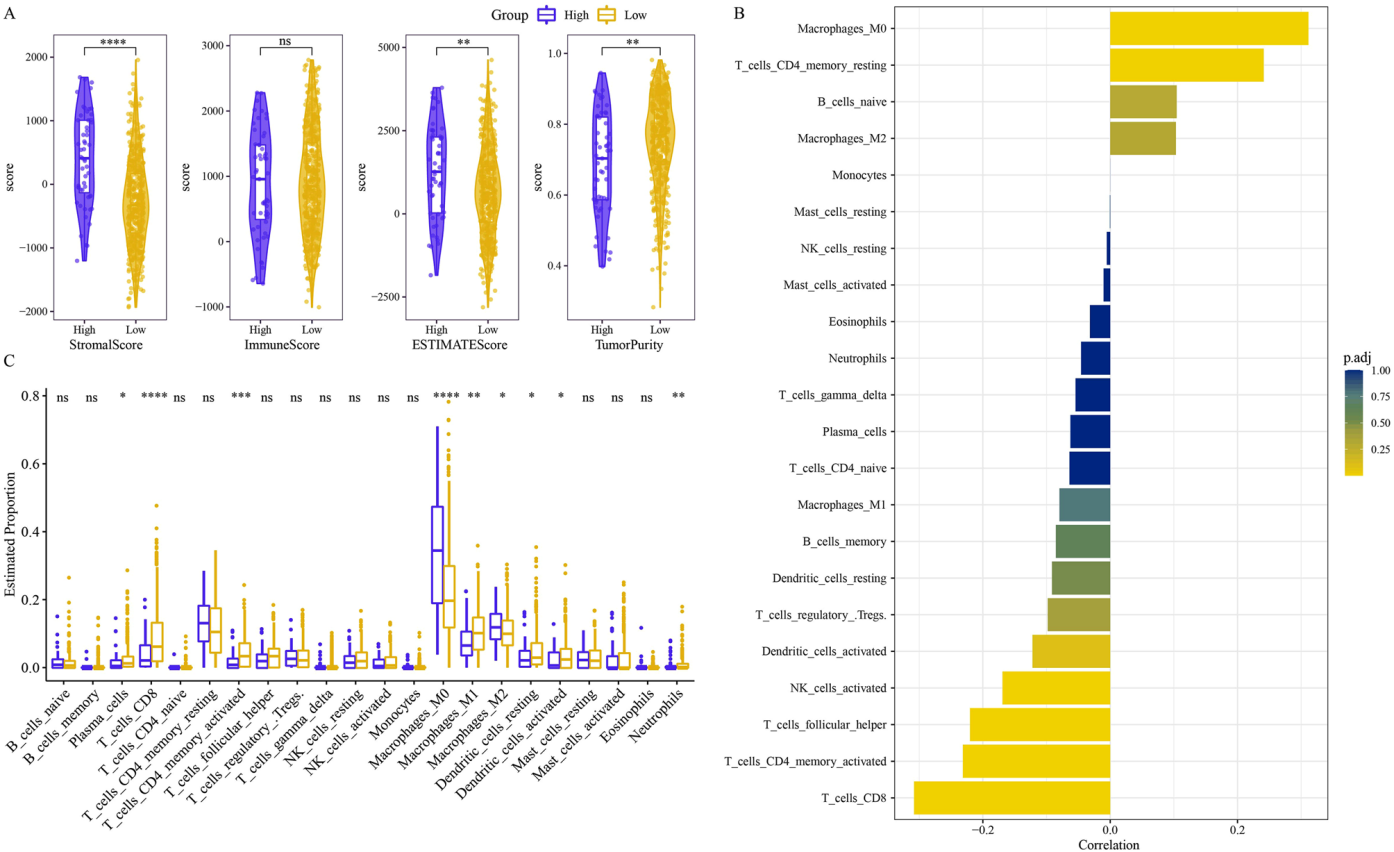
The Hedgehog pathway showed a potential impact on the TME of samples in HNSCC. (**A**) Stromal score, immune score, ESTIMATE score and tumor purity differences between samples with high Hedgehog pathway scores and samples with low Hedgehog pathway scores. (**B**) Correlation coefficient between Hedgehog pathway score and abundance of immune cell infiltration. (**C**) Differences in the abundance of immune cell infiltration between samples with high Hedgehog pathway scores and those with low Hedgehog pathway scores.

We explored whether Hedgehog pathway activity affected the TME. Stromal score and ESTIMATE score calculated by ESTIMATE showed significantly improved levels in samples with high Hedgehog pathway scores compared to samples with low Hedgehog pathway scores. However, tumor purity was completely opposite to the trend of these two scores. Immune score and Hedgehog pathway score were not significantly correlated (Fig. 3A). Hedgehog pathway score was positively correlated with M0 macrophages and resting memory CD4 T cells, and was negatively correlated with activated memory CD4 T cells, CD8 T cells, and helper follicular T cells (Fig. 3B). From the results of the comparison of immune cell infiltration abundance between samples with high Hedgehog pathway scores and samples with low Hedgehog pathway scores, the infiltration abundance of plasma cell, CD8 T cell, M1 macrophage, resting dendritic cell, activated dendritic cell, activated memory CD4 T cell in the samples with low Hedgehog pathway scores were significantly higher than those of the samples with high Hedgehog pathway scores (Fig. 3C).

### Identification of the gene modules most relevant to the Hedgehog pathway

**Figure 4:**
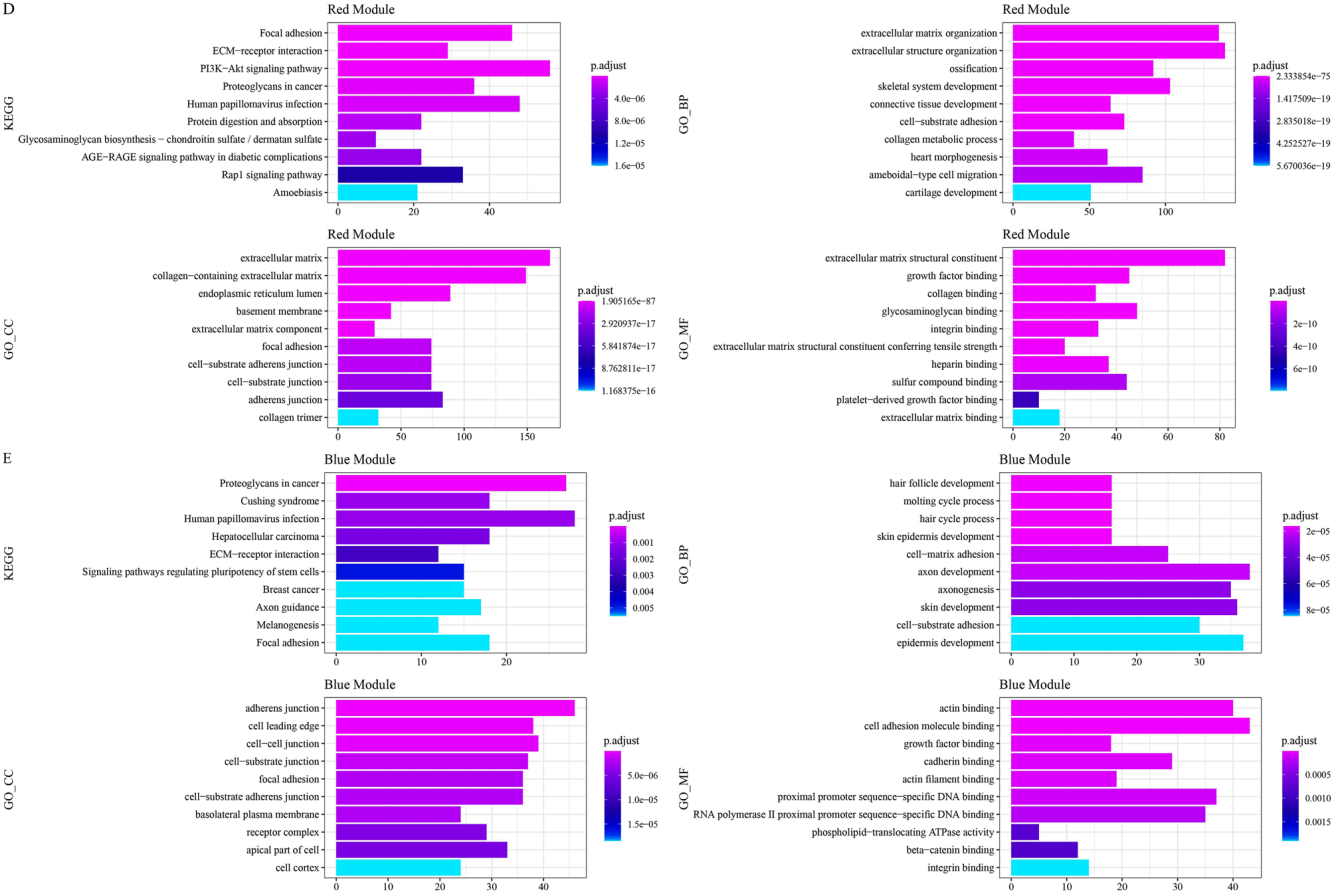
Identification of the gene modules most relevant to the Hedgehog pathway. (**A**) Heatmap of the correlation between module eigengenes (MEs) and clinical traits as well as Hedgehog pathway. (**B**) Scatter plot of the correlation between GS and MM in the red module. (**C**) Scatter plot of the correlation between GS and MM in the bule module. (**D**) KEGG pathways and GO terms with significantly enriched genes in the red modules. (**E**) KEGG pathways and GO terms for significantly annotated genes in the blue module.

A soft threshold of 7 was chosen to build the co-expression network, thereby clustering highly interconnected genes into nine modules (Fig. S1A,B). The red module had the largest number of genes with similar expression patterns and was the most relevant module of Hedgehog pathway, while the blue module was the second most relevant module of Hedgehog pathway (Fig. 4A, Fig. S1C). Correlation coefficient between module membership (MM) and gene significance (GS) in red module reached 0.87, and in bule module reached 0.66 (Fig. 4B,C). Pathways significantly annotated by genes in the red module included focal adhesion, ECM–receptor interaction, PI3K–Akt signaling pathway, proteoglycans in cancer, human papillomavirus infection (Fig. 4D). The genes in bule module were significantly enriched in human papillomavirus infection, proteoglycans in cancer, Hepatocellular carcinoma, Cushing syndrome, etc. (Fig. 4E). Therefore, the red and blue modules were the most relevant gene modules of the Hedgehog pathway, and the pathways regulated by the two modules were partially consistent.

### Formation and validation of the Hedgehog associated 6-gene signature

**Figure 5:**
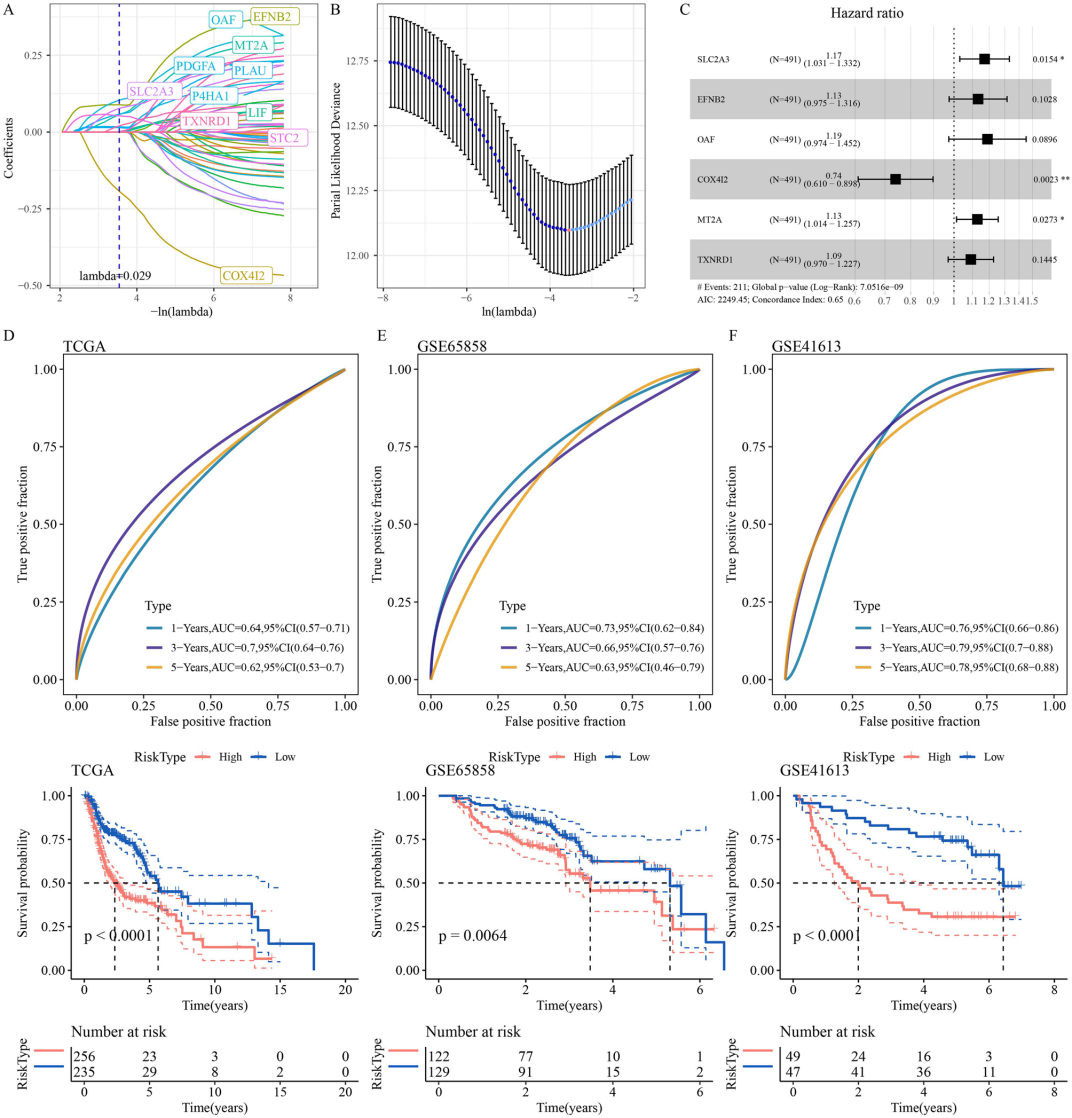
Formation and validation of the Hedgehog associated 6-gene signature. (**A**) Five-fold cross-validation of the LASSO model. (**B**) The plot of the the penalty term parameters, with log(λ) values on the abscissa and degrees of freedom on the ordinate. (**C**) The forest plot shows the hazard ratio and the p value representing prognostic significance for each gene in the model calculated from the multivariate Cox regression analysis. (**D**) ROC curve and Kaplan–Meier curve for prognostic classification of the model in the TCGA-HNSC cohort. (**E**) ROC curve and Kaplan–Meier curve to validate the accuracy of the model in the GSE65858 cohort. (**F**) Performance validation of the model in the GSE41613 cohort: ROC curve and survival curve.

A total of 5288 differentially up-regulated genes were identified between tumor samples and normal samples in the TCGA-HNSC cohort (Fig. S2A), and the intersection of these genes with the most relevant gene modules of the Hedgehog pathway included 783 genes (Fig. S2B). Univariate Cox regression analysis identified 60 genes as prognostic genes with a threshold of p < 0.01. LASSO analysis was conducted to achieve a balanced adjustment of model error and variance by adjusting λ, where λ was chosen to be 0.029 so that 11 genes were included in the generalized linear model (Fig. 5A,B). Stepwise model selection was performed by stepAIC, and the last six genes were included in the risk model (Fig. 5C). Multivariate Cox regression analysis calculated the regression coefficient of each gene, and the model formula was obtained: RiskScore = 0.1583536 * SLC2A3 + 0.1246219 * EFNB2 + 0.1731163 * OAF-0.3015618 * COX4I2 + 0.1212065 * MT2A + 0.0873815 * TXNRD1. The risk scores of samples in TCGA-HNSC, GSE65858 and GSE41613 cohorts were obtained by using the formula to evaluate the prognosis. The accuracy of the model was different in distinct cohorts. The 1 -, 3 -, and 5-year AUC of the model in the TCGA-HNSC cohort were 0.64, 0.7, and 0.62, respectively. There were significant differences in the survival curves and survival statistics between the high-risk group and the low-risk group (Fig. 5D). The ROC curve of GSE65858 cohort showed that the classification efficiency of the model for 1-, 3- and 5-year survival of HNSCC patients was 0.73, 0.66 and 0.63, respectively, and the survival time of high-risk patients was worse than that of low-risk patients (Fig. 5E). The GSE41613 cohort had the highest classification efficiency, with AUC above 0.75 at 1 year, 3 years and 5 years, and the difference in survival trend between high-risk group and low-risk group was the most significant (Fig. 5F).

### Prognostic independence and clinical value of Hedgehog associated signature

**Figure 6:**
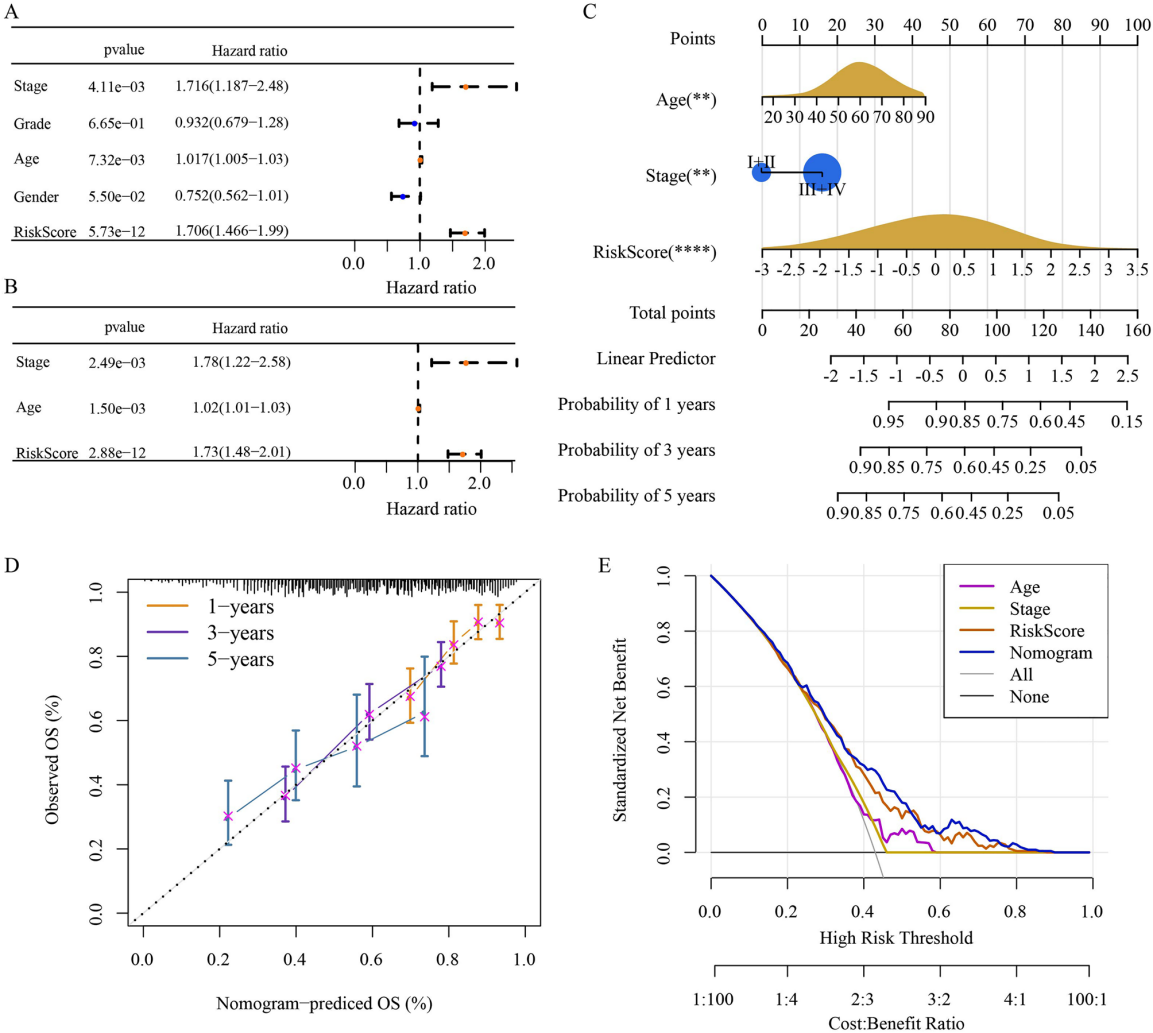
Prognostic independence and clinical value of Hedgehog associated signature. (**A**) Univariate and Cox regression analyses for stage, grade, age, gender and risk score in TCGA-HNSC. (**B**) Multivariate Cox regression analysis. (**C**) Multivariate Cox regression analysis. (**D**) The nomogram consisting of age, stage and risk score. (**E**) The net benefit of nomogram and independent prognostic indicators for predicting OS of HNSCC.

The clinical indicators recorded in the TCGA-HNSC cohort, including stage, grade, age and gender, and Hedgehog associated signature constructed in this study were included in univariate and multivariate Cox regression analyses to determine the independent prognostic indicators: stage, age and risk score (Fig. 6A,B). The nomogram formed by these three indicators showed that the risk score corresponded to the highest points (Fig. 6C). The prediction probability of 1 -, 3 -, and 5-year OS of nomogram was close to the actual probability, and the error of prediction was small (Fig. 6D). The net benefit of the nomogram for predicting OS of HNSCC was also greater than that of the other three independent prognostic indicators (Fig. 6E). Therefore, the nomogram had potential prognostic value for HNSCC.

### Hedgehog associated signature was related to cancer-promoting mechanisms

**Figure 7:**
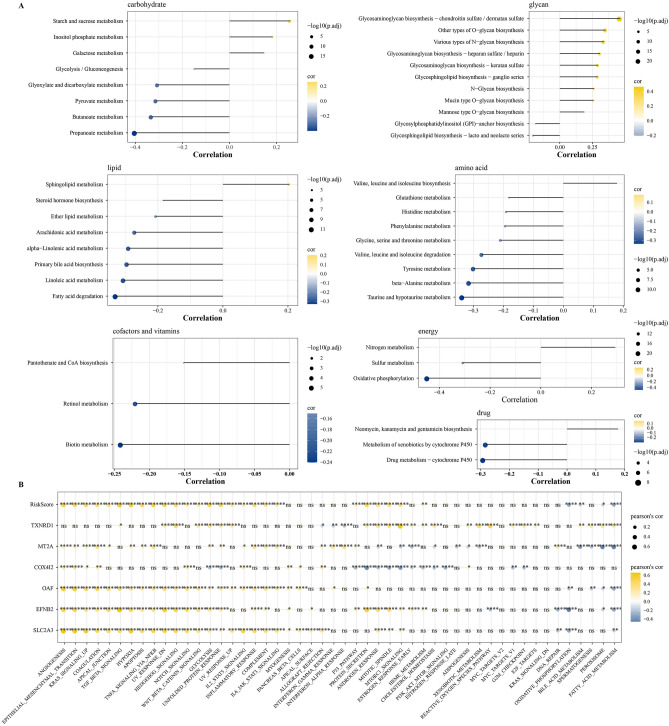
Hedgehog associated signature was related to cancer-promoting mechanisms. (**A**) The association between the risk score calculated by Hedgehog associated signature and the metabolism of different substances. (**B**) The correlation between single gene in risk score as well as Hedgehog associated signature and hallmark pathway.

Since the Hedgehog pathway itself was related to metabolism, the association between the risk score calculated by Hedgehog associated signature and metabolic pathways was analyzed. In carbohydrate metabolism, starch and sucrose metabolism had the strongest positive relation to risk score, while propanoate metabolism had the strongest negative relation to risk score. Most metabolic pathways in Glycan metabolism were significantly positively related to risk score, while most pathways in lipid metabolism and amino acid metabolism were significantly negatively related to risk score. Most metabolic pathways in Glycan metabolism were significantly positively related to risk score, while most pathways in lipid metabolism and amino acid metabolism were significantly negatively correlated with risk score. Retinol metabolism and biotin metabolism in Cofactors, Pantothenate and CoA biosynthesis, and vitamins metabolism were also negatively correlated with risk score. In energy metabolism, nitrogen metabolism had a positive correlation with risk score, while oxidative phosphorylation and sulfur metabolism had a strong negative correlation with risk score. Neomycin and kanamycin and gentamicin biosynthesis in drug metabolism were positively correlated with risk score, whereas drug metabolism–cytochrome P450 and metabolism of xenobiotics by cytochrome P450 had a high negative correlation with risk score (Fig. 7A). In the hallmark pathway, the oncogenic cell pathway, including angiogenesis, epithelial-mesenchymal transition, TGFβ signaling, TNFA signaling via NFκB, hypoxia, inflammatory response and glycolysis were significantly positively correlated with risk score (Fig. 7B).

### Hedgehog associated signature influenced the immune indication and immunotherapy response of HNSCC

**Figure 8:**
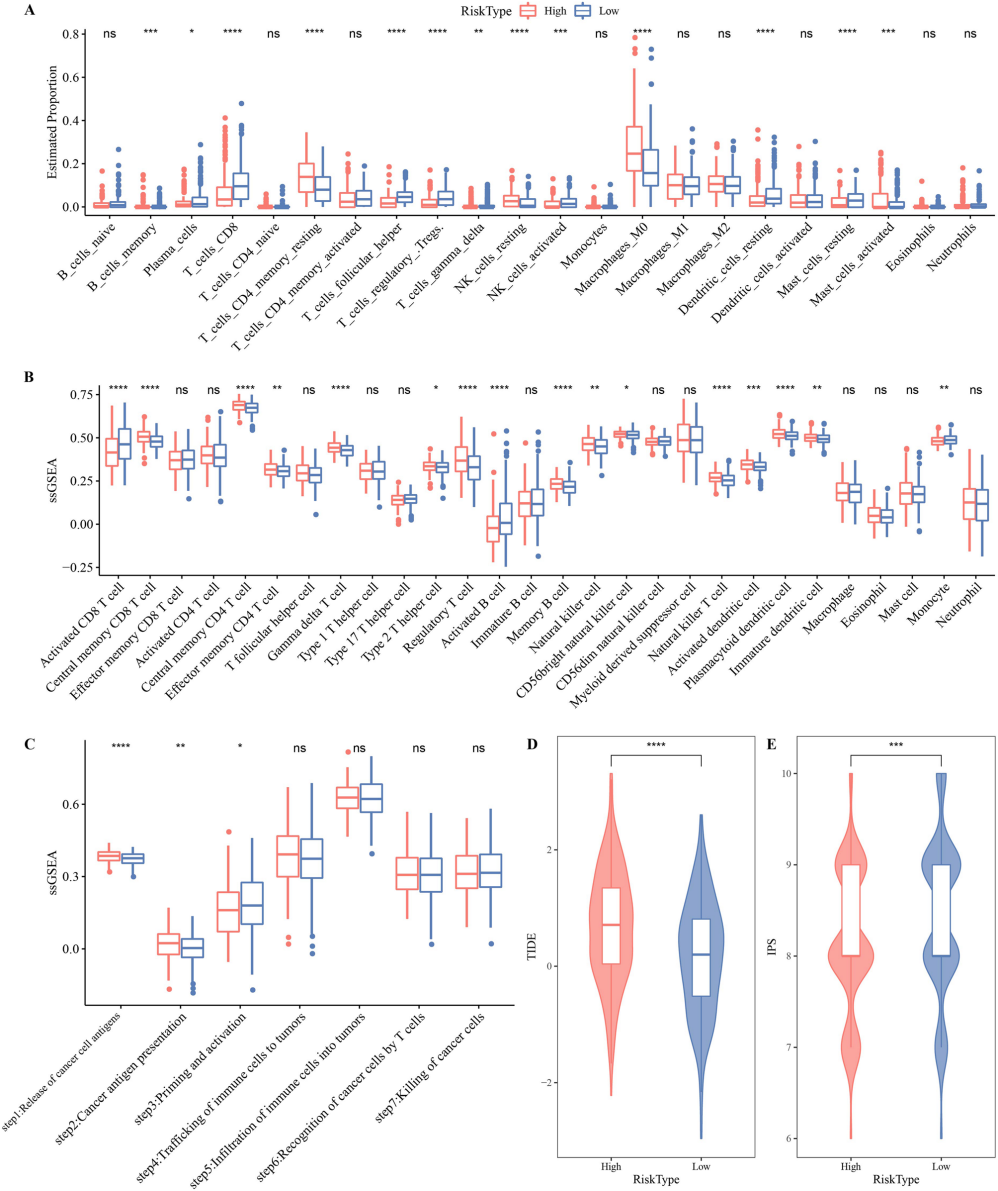
Hedgehog associated signature influenced the immune indication and immunotherapy response of HNSCC. (**A**) Differences in infiltrating abundance of immune cells between the two groups classified according to Hedgehog associated signature. (**B**) Differences in enrichment scores of immune cells between the two groups classified according to Hedgehog associated signature. (**C**) The difference of enrichment degree of seven-step anticancer immune steps between the two groups classified according to Hedgehog associated signature. (**D**) TIDE score differences between the two risk types. (**E**) Differences in IPS between the two risk types.

In Fig. 7B, a significant positive correlation between risk score and immunomodulatory pathways was found, including inflammatory response, complement, IL2/STAT5 signaling, IL6/JAK/STAT3 signaling, interferon α and β response. To gain a more detailed understanding of the immune indications affected by Hedgehog associated signature, differential analysis of immune cell infiltration abundance was performed between high-risk and low-risk samples. Cells occupying a high proportion of the TME, such as CD8 T cells, resting memory CD4 T cells and M0 macrophages, showed significantly different infiltration abundance between the two risk groups. The infiltrating abundance of CD8 T cells was significantly higher in the low-risk group, whereas M0 macrophages and resting memory CD4 T cells were significantly more abundant in the high-risk group (Fig. 8A). The enrichment scores of activated CD8 T cells, activated B cells and monocyte were also significantly higher in the low-risk group. The enrichment scores of other immune cells, including central memory CD4 T cell, central memory CD8 T cell, effector memory CD4 T cell, gamma delta T cell, natural killer cell, natural killer T cell, CD56 bright natural killer cell, activated dendritic cell, plasmacytoid dendritic cell, immature dendritic cell, were significantly higher in the high-risk group (Fig. 8B). The response of steps 1 and 2 in the seven-step anticancer immunization step was stronger in the high-risk group, and the response of step 3 was stronger in the low-risk group (Fig. 8C). The low-risk group had a significantly lower TIDE score and a significantly higher IPS than those with a high risk, indicating that compared with high-risk patients, low-risk patients had a greater chance of responding to immunotherapy (Fig. 8D,E).

## Discussion

Hedgehog signaling is involved in the development of teeth, lips, palate and salivary glands in the head and neck, and the importance of Hedgehog signaling in the occurrence of HNSCC was first reported in 2011^28,29^: high expression of Sonic hedgehog relates significantly to poor OS in patients with head and neck cancer. Later, Gregory et al. found that Hedgehog signaling induces radiation resistance and matrix-driven tumor re-proliferation in HNSCC^30^. Cancer-associated fibroblast-derived exosomes promote HNSCC proliferation and migration through Hedgehog signaling^31^. Immunohistochemical analysis also supports that expression of Hedgehog pathway proteins SMO and GLI was an independent prognostic factor for HNSCC^32^. More aspects of Hedgehog pathway in HNSCC are still not well established.

In this study, it can be determined that the Hedgehog pathway in HNSCC was overactive, was a risk factor in HNSCC, and was significantly positively correlated with most glycan metabolism, cofactors and vitamins, as well as drug metabolism. Several studies have shown that Hedgehog signaling is present in different stages of carcinogenesis in different tumors. For example, Hedgehog activation is in early tumor stages of pancreatic and esophageal cancers and metastatic tumors. Activation of hedgehog signaling in gastric and prostate cancers is associated with increased potential of tissue invasion and metastasis^33^. This study observed that the degree of hedgehog pathway activation was significantly higher in G2 and G3 than in G1, which may imply that the Hedgehog pathway is more involved in carcinogenesis in the G2–G3 stages of HNSCC. In addition to its direct role in regulating cancer cell properties, Hedgehog signaling has also been found to have immunomodulatory effects on TME^34^. The study by Amy et al. found that Hedgehog signaling promotes intratumoral M2 macrophage polarization and inhibits intratumoral CD8+ T cell recruitment^35^. We found that HNSCC patients with high Hedgehog pathway activity had a higher degree of M2 macrophage infiltration and significantly lower CD8+ T cell infiltration than patients with inactive Hedgehog pathway. HH ligand of the Hedgehog pathway in tumor cells activate signaling in the surrounding stroma to provide a favorable microenvironment for tumor growth^10^. Our measurements are consistent with this phenomenon: stromal activity was also significantly higher in patients with high Hedgehog pathway activity than in those with low Hedgehog pathway activity.

In addition to Hedgehog signaling components, understanding the molecules that act at different levels of Hedgehog signaling is becoming increasingly important and may open new perspectives for the optimization of molecular targeting and therapeutic strategies associated with the Hedgehog pathway. We screened modules highly correlated with Hedgehog by WGCNA and screened genes between HNSCC tumor tissues and normal tissues from the modules, and finally captured six genes to combine into an overall model. and finally captured the model of six genes combined into a whole. Six genes were not all characterized in cancer. The prognostic predictive potential of SLC2A3 for HNSCC has been reported, and this molecule also mediates cancer cell proliferation and, migration, and immune responses^36^. EFNB2 was identified as a tumor promoter in HNSCC that maximized tumor size and vascular normalization when knocked down in cancer cells and blood vessels^37^. COX4I2 is highly correlated with the blood supply of adrenal pheochromocytoma and contributes to angiogenesis^38^. High-expressed MT2A is associated with poor esophageal cancer prognosis and induces malignant physiological processes of cancer cells^39^. In neoadjuvant chemoradiation therapy specimens, preoperative TXNRD1 independently predicted overall survival in esophageal squamous cancer patients^40^. In this study, Hedgehog associated 6-gene signature provided more comprehensive predictive information than single molecule and was a prognostic indicator of HNSCC, and whose net benefit was higher than each of the clinical features recorded in TCGA-HNSC. Hedgehog associated 6-gene signature also showed significant positive correlation with angiogenesis, inflammatory response and EMT as Hedgehog pathway. Zhang et al.^41^ identified the gene modules most significantly associated with tumorigenesis in oral squamous cell carcinoma by WGCNA, and the modules dominated intercellular adhesion, extracellular matrix, and collagen catabolism metabolism processes in tumors. And 10 hub genes were identified. Our study provides new insights into the role of the Hedgehog signaling pathway in the progression of head and neck squamous cell carcinoma, and introduces a novel gene signature that contributes to the understanding of pathological progression and immunotherapeutic response in OSCC. These findings not only help to advance the understanding of HNSCC pathogenesis, but also provide a basis for identifying potential therapeutic targets and improving immunotherapy strategies. Moreover, Hedgehog associated 6-gene signature was also significant in predicting the response to immunotherapy in HNSCC, and thus was considered as a predictive biomarker for immunotherapy response in HNSCC.

However, this study still has limitations. First, we identified prognostic factors in HNSCC as well as constructed a prognostic system, albeit with excellent prognostic performance in all three datasets. However, we still lack a clinical independent cohort to further validate the model performance. Future clinical cohort validation with large multi-center samples is needed. Second, future in vitro and in vivo experiments are still needed to explore the functions of the six prognostic factors in HNSCC and their molecular mechanisms in tumor progression.

In summary, the present study clarified the Hedgehog pathway hyperactivity in HNSCC and the impact on HNSCC metabolism and components within the TME. A Hedgehog associated 6-gene signature based on six targets was provided, distinguishing characteristics of patients in different risk groups, including OS, metabolic profile and TME performance and immunotherapy response, with potential implications for future cancer interception.

### Supplementary Information

Supplementary Figure S1.

Supplementary Figure S2.

Supplementary Legends.

## Data Availability

The datasets generated and/or analyzed during the current study are available in the public database, including [GSE65858] repository, [https://www.ncbi.nlm.nih.gov/geo/query/acc.cgi?acc=GSE65858] and [GSE41613] repository, [https://www.ncbi.nlm.nih.gov/geo/query/acc.cgi?acc=GSE41613].

## Abbreviations

HNSCC: Head and neck squamous cell carcinoma
TME: Tumor microenvironment
LASSO: Least absolute shrinkage and selection operator
SMO: Smoothened
TCGA: The Cancer Genome Atlas
GEO: Gene Expression Omnibus
OS: Overall survival
SsGSEA: Single-sample gene set enrichment analysis
WGCNA: Weighted co-expression network analysis
MAD: Median absolute deviation
TOM: Topological overlap matrix
ROC: Receiver operating characteristic
AUC: Areas under the ROC curve
DCA: Decision curve analysis
GSVA: Gene set variation analysis
IPS: Immunophenoscore
TIDE: Tumor immune dysfunction and exclusion
ICB: Immune checkpoint blockade
GS: Gene significance
MM: Module membership

## References

[1] Mody MD, Rocco JW, Yom SS, Haddad RI, Saba NF (2021). Head and neck cancer. Lancet.

[2] Caudell JJ (2022). NCCN guidelines(R) insights: Head and neck cancers, version 1.2022. J. Natl. Compr. Cancer Netw..

[3] Guigay J (2015). The overall treatment of head and neck squamous cell carcinoma (SCC) in 2015. Oncologie.

[4] Runnels J (2023). Combining radiotherapy and immunotherapy in head and neck cancer. Biomedicines.

[5] Even C, Le Tourneau C (2015). Molecular targeted therapies in head and neck cancer’s treatment. Oncologie.

[6] Kaidar-Person O, Gil Z, Billan S (2018). Precision medicine in head and neck cancer. Drug Resist. Update.

[7] Saada E, Ferrand F, Peyrade F, Guigay J (2015). Management of metastatic head and neck squamous cell carcinomas. Oncologie.

[8] Gordon K, Smyk D, Gulidov I, Golubev K, Fatkhudinov T (2023). An overview of head and neck tumor reirradiation: What has been achieved so far?. Cancers (Basel).

[9] Giammona A, Crivaro E, Stecca B (2023). Emerging roles of Hedgehog signaling in cancer immunity. Int. J. Mol. Sci..

[10] Jeng KS, Chang CF, Lin SS (2020). Sonic Hedgehog signaling in organogenesis, tumors, and tumor microenvironments. Int. J. Mol. Sci..

[11] Jiang J (2022). Hedgehog signaling mechanism and role in cancer. Semin. Cancer Biol..

[12] Dlugosz A, Agrawal S, Kirkpatrick P (2012). Vismodegib. Nat. Rev. Drug Discov..

[13] Casey D (2017). FDA approval summary: Sonidegib for locally advanced basal cell carcinoma. Clin. Cancer Res..

[14] Kantarjian H (2021). Harnessing the benefits of available targeted therapies in acute myeloid leukaemia. Lancet Haematol..

[15] Carpenter RL, Ray H (2019). Safety and tolerability of sonic Hedgehog pathway inhibitors in cancer. Drug Saf..

[16] Lemos T, Merchant A (2022). The hedgehog pathway in hematopoiesis and hematological malignancy. Front. Oncol..

[17] Quaglio D, Infante P, Di Marcotullio L, Botta B, Mori M (2020). Hedgehog signaling pathway inhibitors: An updated patent review (2015–present). Expert Opin. Ther. Pat..

[18] Jain R, Dubey SK, Singhvi G (2022). The Hedgehog pathway and its inhibitors: Emerging therapeutic approaches for basal cell carcinoma. Drug Discov. Today.

[19] Liberzon A (2015). The Molecular Signatures Database (MSigDB) hallmark gene set collection. Cell Syst..

[20] Langfelder P, Horvath S (2008). WGCNA: An R package for weighted correlation network analysis. BMC Bioinform..

[21] Ritchie ME (2015). limma powers differential expression analyses for RNA-sequencing and microarray studies. Nucleic Acids Res..

[22] Blanche P, Dartigues JF, Jacqmin-Gadda H (2013). Estimating and comparing time-dependent areas under receiver operating characteristic curves for censored event times with competing risks. Stat. Med..

[23] Wu T (2021). clusterProfiler 4.0: A universal enrichment tool for interpreting omics data. Innovation (Camb.).

[24] Newman AM (2015). Robust enumeration of cell subsets from tissue expression profiles. Nat. Methods.

[25] Bagaev A (2021). Conserved pan-cancer microenvironment subtypes predict response to immunotherapy. Cancer Cell.

[26] Xu L (2018). TIP: A web server for resolving tumor immunophenotype profiling. Cancer Res..

[27] Charoentong P (2017). Pan-cancer immunogenomic analyses reveal genotype-immunophenotype relationships and predictors of response to checkpoint blockade. Cell Rep..

[28] Schneider S (2011). Expression of the Sonic hedgehog pathway in squamous cell carcinoma of the skin and the mucosa of the head and neck. Head Neck.

[29] Cierpikowski P, Leszczyszyn A, Bar J (2023). The role of Hedgehog signaling pathway in head and neck squamous cell carcinoma. Cells.

[30] Gan GN (2014). Hedgehog signaling drives radioresistance and stroma-driven tumor repopulation in head and neck squamous cancers. Cancer Res..

[31] Zhao G (2020). Exosomal sonic Hedgehog derived from cancer-associated fibroblasts promotes proliferation and migration of esophageal squamous cell carcinoma. Cancer Med..

[32] Richtig G (2019). Hedgehog pathway proteins SMO and GLI expression as prognostic markers in head and neck squamous cell carcinoma. Histopathology.

[33] Skoda AM (2018). The role of the Hedgehog signaling pathway in cancer: A comprehensive review. Bosn. J. Basic Med. Sci..

[34] Hanna A, Shevde LA (2016). Hedgehog signaling: Modulation of cancer properties and tumor mircroenvironment. Mol. Cancer.

[35] Petty AJ (2019). Hedgehog signaling promotes tumor-associated macrophage polarization to suppress intratumoral CD8+ T cell recruitment. J. Clin. Investig..

[36] Chai F (2023). Identification of SLC2A3 as a prognostic indicator correlated with the NF-kappaB/EMT axis and immune response in head and neck squamous cell carcinoma. Channels (Austin).

[37] Bhatia S (2022). EphB4 and ephrinB2 act in opposition in the head and neck tumor microenvironment. Nat. Commun..

[38] Sun F (2019). From clinic to mechanism: Proteomics-based assessment of angiogenesis in adrenal pheochromocytoma. J. Cell. Physiol..

[39] Shimizu M (2021). Metallothionein 2A expression in cancer-associated fibroblasts and cancer cells promotes esophageal squamous cell carcinoma progression. Cancers (Basel).

[40] Akaishi R (2022). Correlation between TXNRD1/HO-1 expression and response to neoadjuvant chemoradiation therapy in patients with esophageal squamous cell carcinoma. Esophagus.

[41] Zhang X (2018). Application of weighted gene co-expression network analysis to identify key modules and hub genes in oral squamous cell carcinoma tumorigenesis. Onco Targets Ther..

