# Supplementary figures and images for: Elucidating Hedgehog pathway's role in HNSCC progression: insights from a 6-gene signature
Source: Sci Rep. 2024 Feb 26;14:4686. doi: 10.1038/s41598-024-54937-6 (PMC10897175; doi:10.1038/s41598-024-54937-6)

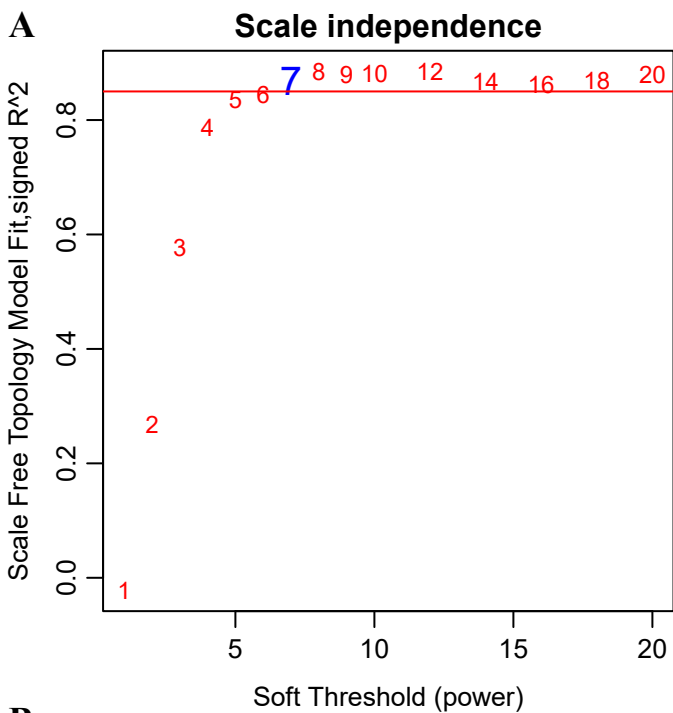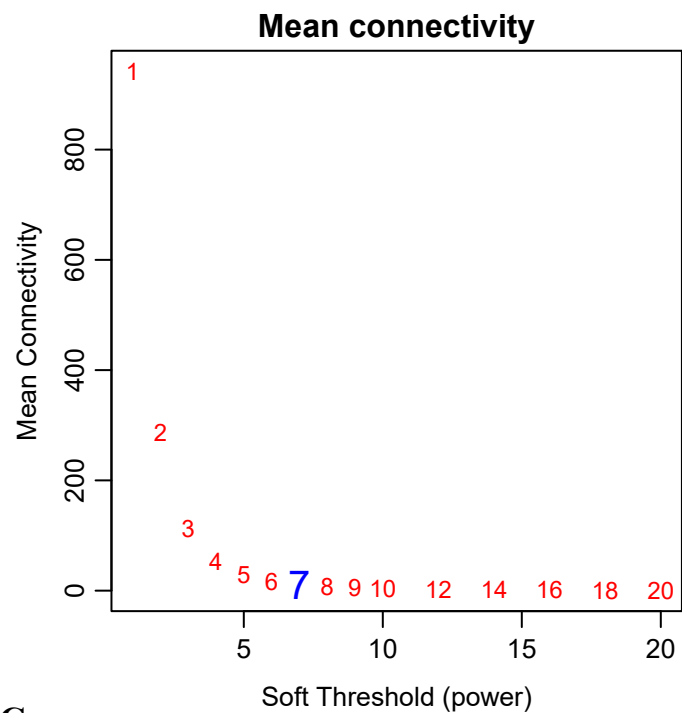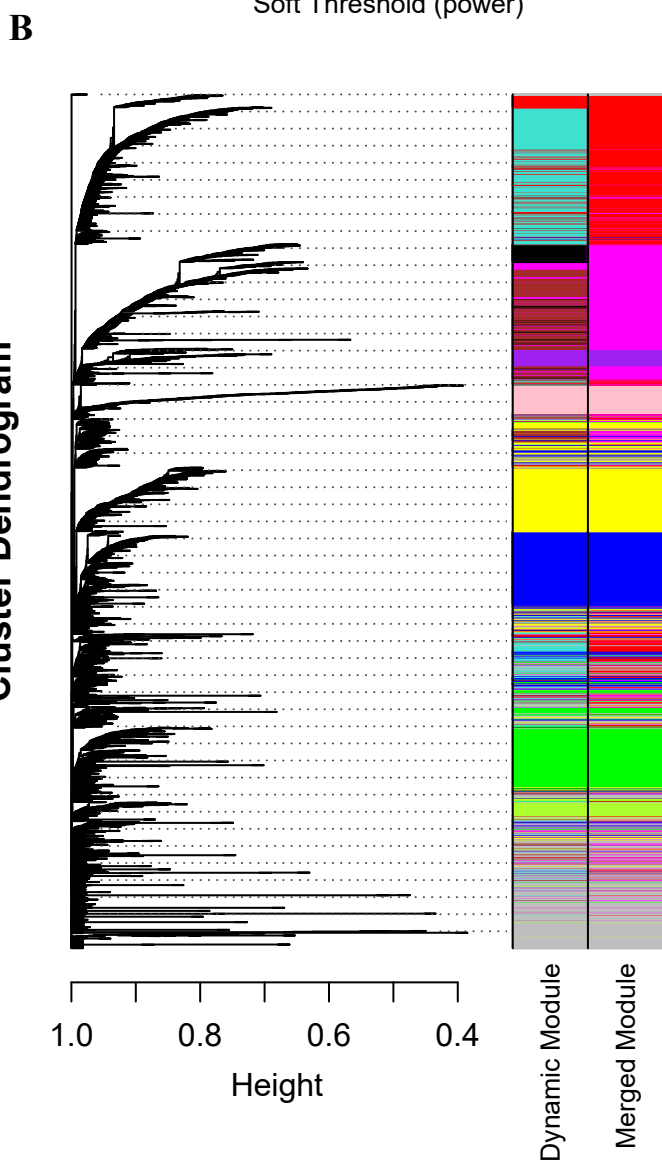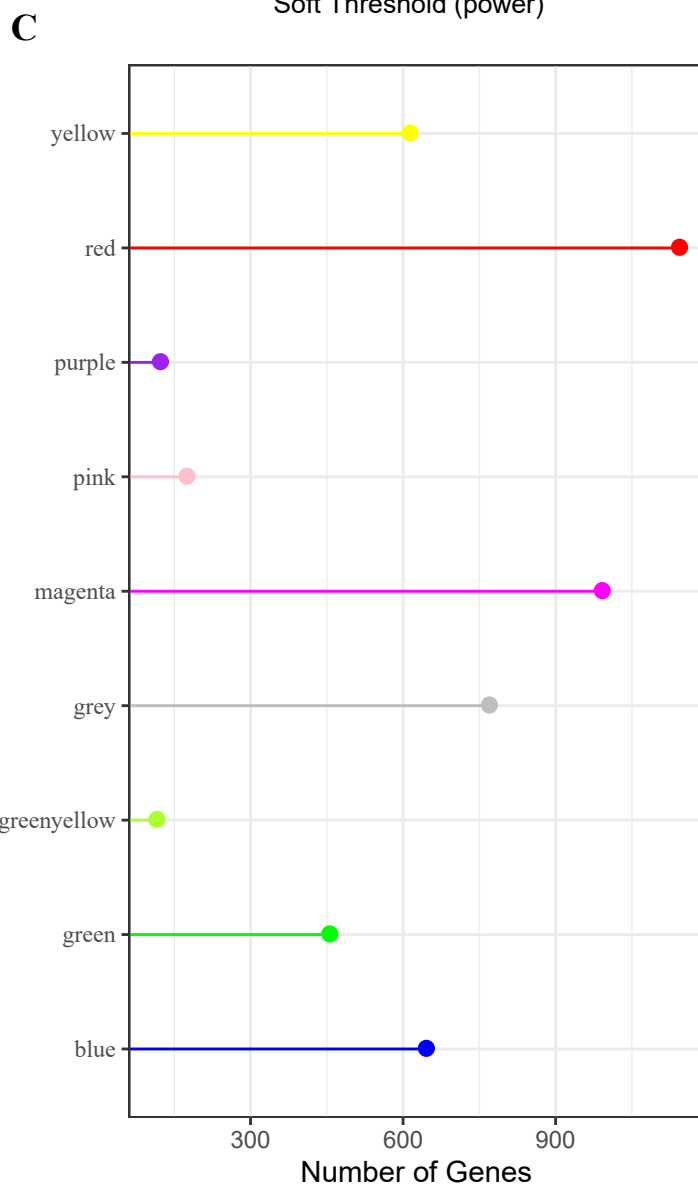

Supplement: Supplementary file 1 — Supplementary Figure S1. [file 41598_2024_54937_MOESM1_ESM.pdf]

**A**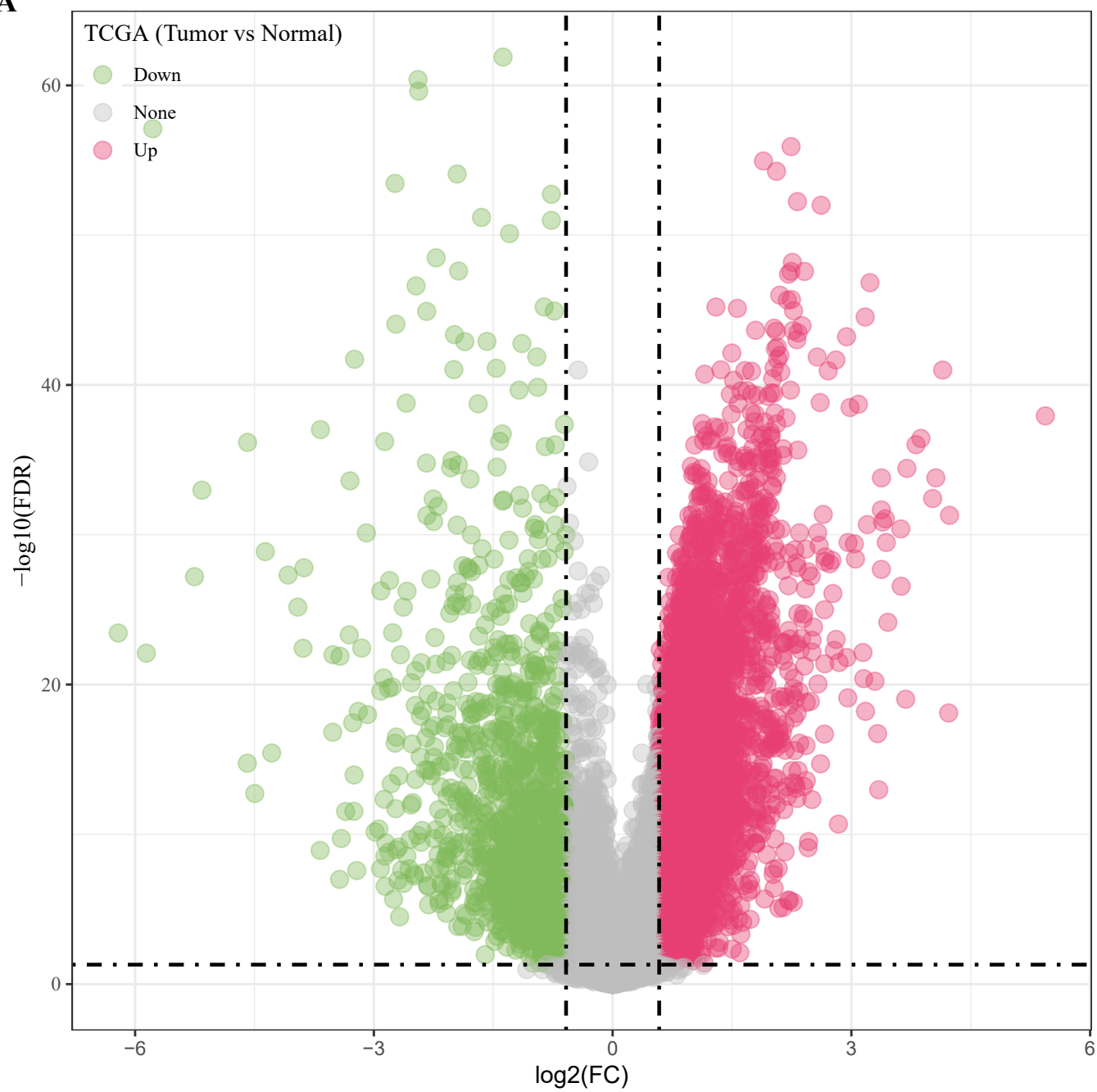**B**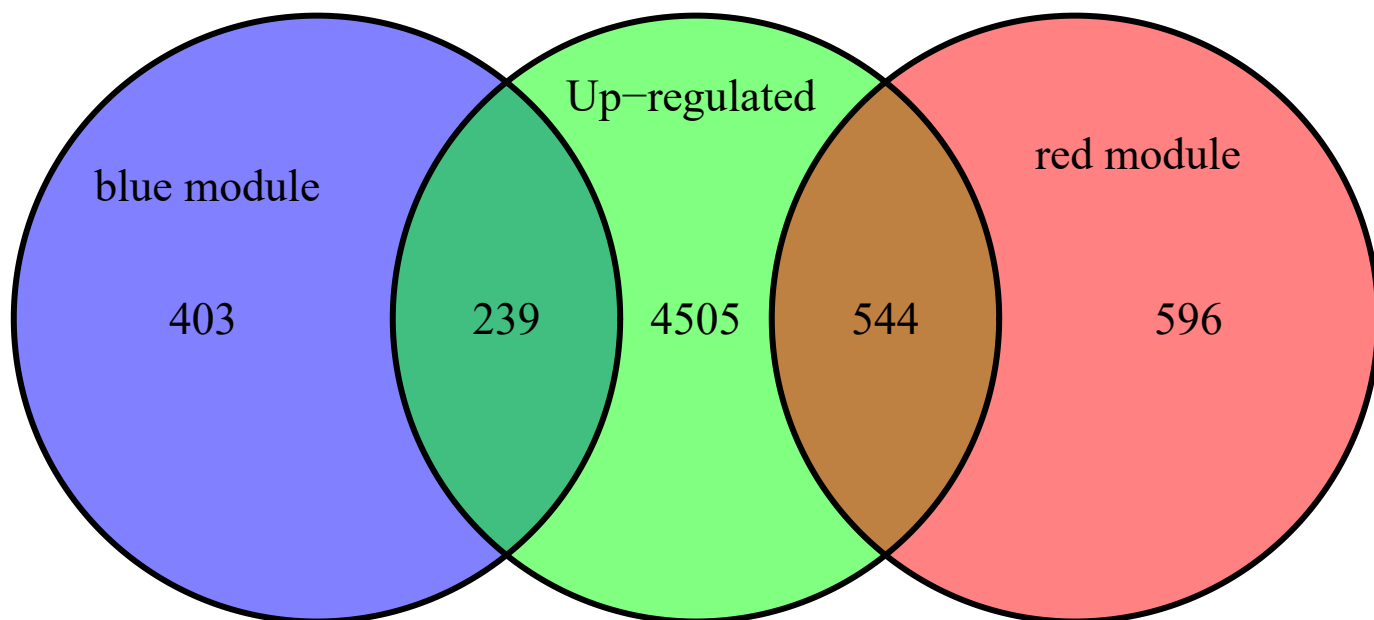

Supplement: Supplementary file 2 — Supplementary Figure S2. [file 41598_2024_54937_MOESM2_ESM.pdf]
