# Supplementary material for: Elucidating Hedgehog pathway's role in HNSCC progression: insights from a 6-gene signature
Source: Sci Rep. 2024 Feb 26;14:4686. doi: 10.1038/s41598-024-54937-6 (PMC10897175; doi:10.1038/s41598-024-54937-6)
Supplement: Supplementary file 3 — Supplementary Legends. [file 41598_2024_54937_MOESM3_ESM.docx]

**­­Legend of Supplementary Figures**

**Figure S1 WGCNA**

(A) Scale independence and mean connectivity of distinct soft-thresholding powers (β) from 1 to 20. (B) Cluster dendrogram shows modules formed by clusters of genes with similar expression patterns. (C) The number of genes with similar expression patterns in each module.

**Figure S2** **Screening of Hedgehog pathway-related genes**

(A) Volcano plot of the difference analysis between tumor samples and normal samples from the TCGA-HNSC cohort. (B) Venn diagram shows the intersection of the 5288 differentially upregulated genes with the red and blue modules.
